# Effects of pemafibrate (a selective PPAR-α agonist) versus weight loss through an intensive lifestyle modification on liver enzymes, serum soluble dipeptidyl peptidase-4, and hepatic steatosis and fibrosis evaluated by FibroScan transient elastography in people with type 2 diabetes and MASLD

**DOI:** 10.1016/j.clinsp.2025.100839

**Published:** 2025-11-13

**Authors:** Kanako Kato, Teruo Jojima, Dai Tanuma, Kanako Suda, Shintaro Sakurai, Toshie Iijima, Takuya Tomaru, Isao Usui, Yoshimasa Aso

**Affiliations:** Department of Endocrinology and Metabolism, Dokkyo Medical University, Mibu, Tochigi, Japan

**Keywords:** MASLD, Selective PPAR-α Agonist, Pemafibrate, Weight loss, FibroScan, sDPP-4/CD26, Type 2 diabetes

## Abstract

**Objective:**

The present study aimed to investigate the effects of pemafibrate (a selective proliferator-activated receptor-α agonist) vs. weight loss through intensive lifestyle modification on hepatic steatosis and fibrosis evaluated by FibroScan and on serum levels of soluble Dipeptidyl Peptidase-4 (sDDP-4)/CD26, a hepatokine, in people with type 2 diabetes and Metabolic dysfunction-Associated Steatotic Liver Disease (MASLD).

**Methods:**

In a randomized, active-controlled, open-label trial, 60 patients with type 2 diabetes and MASLD were randomized to receive 24 weeks’ treatment with pemafibrate (0.2 mg/day; n=24) or an Intensive Lifestyle Intervention (ILI group; *n* = 27). FibroScan transient elastography was used to assess hepatic steatosis and fibrosis by Controlled Attenuation Parameter (CAP) and Liver Stiffness Measurement (LSM), respectively. Serum levels of sDPP-4/CD26 were measured with a commercial ELISA kit.

**Results:**

Body weight decreased significantly in the ILI group but not in the pemafibrate group. At week 24, CAP showed a significant decrease in the ILI group but no significant change in the pemafibrate group. LSM was unchanged in both groups. Serum Alanine Aminotransferase (ALT), γ-glutamyl transpeptidase, and sDPP-4/CD26 levels decreased significantly in both groups. Changes in ALT during treatment with pemafibrate were positively correlated with changes in serum sDPP-4 and the FAST score.

**Conclusions:**

In people with type 2 diabetes and MASLD, although weight loss through an ILI was better than pemafibrate in terms of the main indicators that assess improvements in the pathogenesis of MASLD, such as CAP, pemafibrate is as effective as moderate weight loss for decreasing liver enzymes or serum sDPP-4/CD26.

## Highlights

• Pemafibrate did not improve hepatic steatosis, while mild weight loss improved it.
• Both pemafibrate and mild weight loss decreased serum ALT and GGT levels similarly.
• Both pemafibrate and weight loss showed a similar reduction in serum sDPP-4 levels.
• Weight loss was better than pemafibrate regarding the main pathogenesis of MASLD.
• Pemafibrate may be another therapy for MASLD in type 2 diabetes on lifestyle changes.

## Introduction

Metabolic dysfunction-Associated Steatotic Liver Disease (MASLD), previously termed Non-Alcoholic Fatty Liver Disease (NAFLD), is defined as steatotic liver disease in the presence of one or more cardiometabolic risk factors and the absence of harmful alcohol intake.^1^ The spectrum of MASLD includes steatosis, Metabolic Dysfunction-Associated Steatohepatitis (MASH, previously NASH), fibrosis, cirrhosis, and MASH-related Hepatocellular Carcinoma (HCC). MASLD is a common condition that has become a serious public health problem worldwide.^1^ Type 2 diabetes is clearly and closely related to MASLD, or MASH.^2^

Weight loss through dieting and exercise is the most effective treatment and the mainstay of MASLD.^3^ The guideline of the American Association for the Study of Liver Diseases recommends a weight loss of 3 %‒5 % to improve hepatic steatosis in people with MASLD.^3^ Several studies have demonstrated that sustained and modest weight loss of about 5 % of the initial body weight by lifestyle intervention can reduce hepatic steatosis and liver enzymes in people with type 2 diabetes.^4^ Accumulating evidence has shown that weight loss, whether achieved by lifestyle interventions (dieting and exercise), bariatric surgery, or pharmacotherapy, can improve biomarkers of MASLD as well as prevent progression and, in some cases, reverse fibrosis.^4^

Peroxisome Proliferator-Activated Receptor-α (PPARα) is involved in metabolic homeostasis through regulation of fatty acid transport and peroxisomal and mitochondrial β-oxidation.^5^^,^^6^ By enhancing mitochondrial β-oxidation in the liver, PPARα decreases the fatty acid content and the hepatic production of very low-density lipoprotein,^7^ leading to a reduction in Triglyceride (TG)-rich lipoprotein and TG levels in sera.^8^ Consequently, fibrates, which are PPARα agonists, can decrease TG accumulation in the liver and are assumed to have a protective effect in MASLD. However, some fibrates, such as fenofibrate (Tricor), can increase the risk of liver dysfunction and increase serum liver enzymes in a dose-dependent manner.^9^^,^^10^ Pemafibrate was the first selective PPARα modulator developed to selectively activate transcription of the PPARα target gene.^11^^,^^12^ Several studies demonstrated that pemafibrate significantly reduces serum Alanine Aminotransferase (ALT) and γ-Glutamyl Transpeptidase (γ-GTP) in people with type 2 diabetes and/or MASLD,^13^, ^14^, ^15^, ^16^ suggesting that it has efficacy and safety in the liver and has the potential to improve liver damage due to MASLD.

Dipeptidyl Peptidase-4 (DPP-4) is a 110-kDa cell surface type II transmembrane protein that displays catalytic activity as a serine protease and rapidly cleaves the N-terminal dipeptides of incretin hormones (GLP-1 and GIP). The soluble form of DPP-4/CD26 (sDPP-4/CD26), which lacks the cytoplasmic tail and transmembrane domain, circulates in the blood and possesses DPP-4 enzymatic activity.^17^ Recently, athe present study and several others reported an elevated level of serum sDPP-4/CD26 in patients with type 2 diabetes and MASLD or MASH, ,^18^, ^19^, ^20^ suggesting that serum sDPP-4/CD26 may be considered a new biomarker for liver fibrosis and progressive MASH, especially cirrhosis. Previous studies demonstrated that weight loss achieved by administration of SGLT2 inhibitors or short-term hospitalization with calorie restriction can reduce serum sDPP-4/CD26 in people with type 2 diabetes and MASLD.^21^^,^^22^ In addition, sDPP-4/CD26 itself may also play a role in crosstalk between the liver and adipose tissue as a hepatokine, since a recent study performed in mice showed that obesity stimulated hepatocytes to produce and secrete sDPP-4, which promoted adipose tissue inflammation and insulin resistance in the liver.^23^ Therefore, reducing the serum level of sDPP-4/CD26 may contribute to an improvement in liver dysfunction in patients with type 2 diabetes and MASLD.

The authors hypothesized that pemafibrate would reduce liver enzymes and hepatic steatosis or fibrosis to the same extent as an intensive lifestyle Intervention (ILI) to induce 3 %‒5 % weight loss, and that pemafibrate reduces the serum sDPP-4/CD26 level in association with liver enzymes in people with type 2 diabetes and MASLD. In this pilot randomized study, the authors therefore compared the effects of 24-weeks of pemafibrate at the 0.2mg dose with an ILI comprised of energy restriction plus exercise designed to induce ∼5 % weight loss on hepatic steatosis and stiffness measured by FibroScan (a transient elastography), serum liver enzymes, and serum sDPP-4/CD26 in people with type 2 diabetes and MASLD.

## Materials and methods

### Participants

The authors studied 60 patients with type 2 diabetes and MASLD who had been referred to the diabetes outpatient clinic of Dokkyo Medical University Hospital, Mibu, Tochigi, Japan. Patients were eligible for enrollment if they had type 2 diabetes and MASLD, were at least 20 years old, and had a glycated Hemoglobin (HbA1c) level of 6.0 % to 12.0 % on stable therapy with 1 to 3 oral antidiabetic agents with or without insulin for at least 3 months.

The diagnosis of MASLD was made on the basis of liver dysfunction (defined as a persistently elevated ALT value greater than or equal to the upper limit for the laboratory), the presence of fatty liver on ultrasonography, low daily alcohol intake (< 30 g for men and < 20 g for women), and exclusion of other liver diseases, such as chronic hepatitis B and C, autoimmune hepatitis, primary biliary cirrhosis, hemochromatosis, and Wilson’s disease. All patients provided written informed consent to participate in the study.

### Study design

This was a randomized, active-controlled, open-label study. Patients were randomly allocated in a 1:1 ratio to receive either 24-weeks’ treatment with pemafibrate (0.2mg/day) or Intensive Lifestyle Intervention (ILI) without any fibrates. Throughout the study, all patients received standard-of-care treatment for type 2 diabetes, and the investigators were encouraged to manage their patients according to local guidelines to achieve optimal glycemic control. The IRI group was individually counseled by dieticians and physical trainers, and provided with written energy restriction plans designed to reduce intake by ∼400 kcal/d plus a supervised moderate-intensity aerobic exercise (150‒200 min/week) over 24 weeks. Participants assigned to the ILI were encouraged to lose at least 5 % of their initial weight at 6 months through a combination of moderate caloric restriction (1200–1500 kcal/day for those individuals weighing < 114 kg and 1500–1800 kcal/day for those weighing > 114 kg, with < 30 % calories from fat and < 10 % from saturated fat) and increased physical activity, with a goal of 175 minutes of moderate intensity physical activity per week. During 6-months, participants in the ILI group attended monthly meetings with both registered dietitians and diabetes specialist nurses. The pemafibrate group received standard-of-care treatment for type 2 diabetes. The dose of the antihypertensive or antihyperglycemic agents, or statins, was not changed from 12 weeks before the start to the end of the study.

This clinical prospective study adheres to the guidelines of the CONSORT Statement.

### Ethics

All interventions in the current study were conducted in adherence to the Declaration of Helsinki in 1964 and its subsequent amendments. The study was approved by the Institutional Review Board of Dokkyo Medical University (C-296–01) and registered with the University Hospital Medical Information Network (UMIN) Clinical Trials Registry (UMIN000044389). All patients gave their written informed consent.

### Assessment methods

Transient elastography was performed with a FibroScan device with the standard 3.5 MHz M probe (Echosens, Paris, France), which measured CAP and LSM simultaneously in the same cylinder of liver parenchyma.^24^ CAP is a measure of ultrasonic attenuation at 3.5 MHz on the FibroScan signal; it is used to assess the severity of liver steatosis and is expressed in dB/m. FibroScan simultaneously assesses LSM by measuring the propagation of an elastic shear wave through the liver parenchyma; LSM is expressed in kPa, with higher values indicating greater stiffness.^24^ The authors used the AST, CAP, and LSM values to calculate the FAST score, as follows^25^: FAST=(e−1.65+1.07×In(LSM)+2.66*10_8×CAP3−63.3×AST_1)/(1+e−1.65+1.07×In(LSM)+2.66*10_8×CAP3−63.3×AST_1) A scan failure was defined as the inability to obtain 10 valid measurements in a single patient.

Visceral Fat Volume (VAT) was measured by dual bioelectrical impedance analysis with a Dual Scan® (Omron Healthcare Company, Limited, Kyoto, Japan). This instrument calculates the cross-sectional area of intra-abdominal fat at the umbilicus from electrical potentials, which are generated by applying small electrical currents at two different locations on the body.^24^ VAT measured by dual bioelectrical impedance is equal to that measured by abdominal computed tomography, the gold standard for determining VAT.^26^ The intra- and inter-assay coefficients of variation of VAT were 6.3 % and 6.8 %, respectively. The person performing and evaluating the FibroScan assessment was blinded to the treatment group.

The serum level of sDPP-4/CD26 was measured with an enzyme-linked immunosorbent assay kit (Human sCD26 Platinum ELISA, Bender MedSystems GmbH, Vienna, Austria). The intra- and inter-assay coefficients of variation were 4.6 % and 9.1 %, respectively.^18^

Serum high molecular weight adiponectin was measured by sandwich enzyme-linked immunosorbent assay with a monoclonal antibody targeting human high molecular weight adiponectin, as described previously.^27^

Serum leptin was measured by radioimmunoassay (Human Leptin RIA Kit, EMD Millipore Co., Billerica, MA, USA).

Serum Remnant Lipoprotein Cholesterol (RLP-C), a TG-rich lipoprotein, was measured with a mixed immunoaffinity gel containing monoclonal anti-human apoA-I (H-12) and anti-human apoB-100 (JI-H) antibodies (Japan Immunoresearch Laboratories, Takasaki, Japan).

Plasma Plasminogen Activator Inhibitor-1 (PAI-1) was measured with a Latex Photometric Immunoassay (LPIA tPAI-1 test, IATRON Laboratories, Tokyo, Japan) that detected both active and latent PAI-1 and PAI-1 bound to tissue plasminogen activator. The intra- and inter-assay coefficients of variation were 2.01 % and 2.38 %, respectively.

The plasma concentration of plasmin-α2-anti-plasmin complex was determined by sandwich enzyme immunoassay (Enzygnost PAP Micro; Dade Behring, Marburg, Germany).

### Outcomes

The primary endpoint was the change in CAP or LSM from baseline to 24 weeks. The main secondary endpoint was the change in liver enzymes (AST, ALT, and γ-Glutamyl Transpeptidase [GGT]) from baseline to 24 weeks, and additional secondary endpoints were the change in HbA1c, VAT, and serum sDPP-4/CD26 levels.

### Statistical analysis

Data are expressed as the mean ± SD or as the median with interquartile range. Differences between groups were analyzed by Student’s paired *t*-test or the unpaired *t*-test, and between-group differences in non-parametric data were analyzed by Wilcoxon’s matched-pairs test or the Mann-Whitney *U* test. Correlations were determined by linear regression analysis or the Spearman rank correlation test. Statistical analyses were performed with GraphPad Prism 7 software (GraphPad Software, Inc., La Jolla, CA), and a p-value of less than 0.05 was accepted as indicating statistical significance.

The authors calculated that a sample of 60 patients was required to detect a difference in the mean CAP of 25 % with 90 % power at a significance level of 0.05 and by assuming an SD of 30.^22^

## Results

**Fig. 1:**
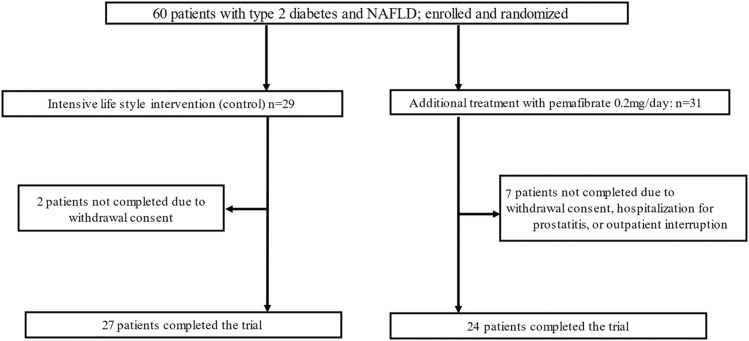
Patients deposition.

A total of 60 patients were screened and randomized to receive pemafibrate (*n* = 31) or standard lifestyle recommendations (*n* = 29; Fig. 1). In the pemafibrate group, 24/31 patients completed the trial, and in the control group, 27/29 patients. At baseline, the two groups were well balanced with respect to demographic characteristics and laboratory data (Table 1).

**Table 1:** Baseline demographic, clinical, and laboratory data for patients with type 2 diabetes treated with pemafibrate (0.2 mg/day) or a standard lifestyle treatment (ILI group).

	Pemafibrate	ILI	p-value
N (M/F)	24 (13/11)	27 (12/15)	0.5793
Age (years)	57.2 ± 13.3	62.7 ± 12.1	0.1270
Body weight (Kg)	71.0 ± 12.3	75.2 ± 12.7	0.3178
BMI (Kg/m^2^)	27.9 ± 3.9	28.6 ± 5.2	0.5954
Waist circumference (cm)	97.5 ± 9.7	98.8 ± 11.1	0.6601
VFA (cm^2^)	118.1 ± 31.9	134.1 ± 55.6	0.2213
SBP (mmHg)	128.1 ± 13.1	132.3 ± 15.9	0.3231
DBP (mmHg)	76.3 ± 7.4	77.0 ± 11.6	0.8164
FPG (mg/dL)	135.1 ± 20.7	138.8 ± 33.4	0.6420
HbA1c ( %)	7.0 ± 0.5	7.3 ± 0.7	0.1409
LDL cholesterol (mg/dL)	119.5 ± 28.9	95.3 ± 32.7	0.0077
Triglyceride (mg/dL)	147.5 (113.3, 207)	131 (93.0, 193)	0.2999
HDL-cholesterol (mg/dL)	50.1 ± 10.6	52.8 ± 12.5	0.4102
RLP-C (mg/dL)	5.65 (4.63, 10.9)	6.4 (3.7, 7.53)	0.5091
AST (U/L)	23 (20, 45)	23 (20, 39)	0.9217
ALT (U/L)	35 (23.3, 47)	30 (19, 46)	0.3755
GGT (U/L)	43 (27.3, 83.5)	36 (23, 69)	0.2988
Uric acid (mg/dL)	6.2 ± 1.7	5.2 ± 1.4	0.0292
eGFR (ml/min/1.73m^2^)	79.9 ± 23.6	75.4 ± 20.9	0.4801
C-reactive protein (mg/dL)	0.1 (0.03, 0.30)	0.13 (0.03, 0.31)	0.6092
HMW adiponectin (μg/mL)	1.47 (0.61, 1.90)	1.75 (0.76, 2.63)	0.3086
Leptin (ng/mL)	21.8 (10.2, 37.4)	17.6 (7.6, 32.6)	0.7397
PAI-1 (ng/mL)	85.2 ± 34.4	76.0 ± 30.1	0.3136
PAP (μg/mL)	0.55 (0.5, 0.78)	0.6 (0.5, 0.8)	0.9356
sDPP-4/CD26 (ng/mL)	1124±250.4	1084±381.8	0.6686
CAP (dB/m)	314.5 ± 35.6	317.6 ± 50.0	0.8072
LSM (kPa)	5.5 (4.25, 8.78)	5.3 (3.48, 8)	0.3226
FAST	0.18 (0.09, 0.50)	0.20 (0.06, 0.52)	0.7802
SGLTi2/ Met/ DPP-4i/ SU/ αGI/ Ins/ GLP	14/ 15/ 5/ 13/ 3/ 6/ 3	13/ 23/ 10/ 20/ 7/ 8/ 4	0.1893
ARB+ACEI/ CCB/ Diuretics	11/ 10/ 0	18/ 12/ 2	0.4361
Statin, n ( %)	15 (60.0)	18 (66.7)	0.7743

Data are the mean±SD or the median and inter-quartile ranges.

BMI, Body Mass Index; VFA, Visceral Fat Area; SCT, Subcutaneous Adipose Tissue; FPG, Fasting Plasma Glucose; Hb, Hemoglobin; HOMA-IR, Homeostasis Model Assessment of Insulin Resistance; LDL, Low-Density Lipoprotein; HDL, High-Density Lipoprotein; AST, Aspartate Aminotransferase; ALT, Alanine Transaminase; GGT, γ-Glutamyltranspeptidase; eGFR, Estimated Glomerular Filtration; Rate; HMW, High Molecular Weight; PAI-1, Plasminogen Activator Inhibitor-1; PAP, Plasmin-α2-Antiplasmin complex; CAP, Controlled Attenuation Parameter; LSM, Liver Stiffness Measurement; FAST, FibroScan-AST; SGLT2i, Sodium Glucose co-Transporter Inhibitor-2 inhibitor; Met, metformin (Glucophage); DPP-4i, Dipeptidyl Peptidase-4 inhibitors; SU, Sulfonylurea; Ins, Insulin; GLP, Glucagon-Like Peptide-1 receptor agonists; ARB, Angiotensin II Receptor Blocker; ACEI, Angiotensin-Converting Enzyme Inhibitor; CCB, Calcium Channel Blocker.

At 24 weeks, CAP showed no significant decrease in the pemafibrate group, but it decreased significantly in the control group. LSM showed no significant change in either group. The FAST score showed a significant decrease at 24 weeks in the control group but not in the pemafibrate treatment group. At 24 weeks, there was a significant decrease in ALT and GGT in both groups (Table 2); however, the reduction in GGT was significantly larger in the pemafibrate group than in the ILI group.

**Table 2:** Changes in clinical parameters in type 2 diabetic patients treated with pemafibrate or with an intensive lifestyle Intervention (ILI).

	Pemafibrate			ILI			Difference b/ w groups
	Baseline	Week 24	p-value	Baseline	Week 24	p-value	p-value
Body weight (Kg)	71.0 ± 12.3	70.4 ± 12.2	0.7118	75.3 ± 17.1	73.9 ± 17.1	0.0021	0.0164
BMI (kg/m^2^)	27.9 ± 3.9	27.9 ± 4.0	0.9398	28.6 ± 5.2	28.4 ± 5.2	0.0108	0.0534
Waist circumference (cm)	97.5 ± 9.7	95.0 ± 6.0	0.0911	98.8 ± 11.1	98.8 ± 12.6	0.2906	0.5710
VFA (cm^2^)	118.1 ± 31.9	122.8 ± 27.62	0.4776	125 (104, 169)	115.5 (90.5, 155)	0.1494	0.0353
SBP (mmHg)	128.1 ± 13.1	127.8 ± 10.8	0.8980	132.3 ± 15.9	133.5 ± 16.4	0.7243	0.2934
DBP (mmHg)	76.6 ± 7.7	75.6 ± 7.8	0.8215	77.0 ± 11.2	76.2 ± 10.3	0.8829	0.1655
FPG (mg/dL)	135.1 ± 20.7	139.8 ± 35.7	0.5686	138.8 ± 33.4	146.4 ± 34.2	0.3001	0.8997
HbA1c ( %)	7.03±0.51	7.30±0.91	0.0622	7.3 ± 0.7	7.4 ± 0.9	0.6117	0.1376
LDL-C (mg/dL)	119.5 ± 28.5	113.1 ± 27.4	0.2186	95.3 ± 32.7	93.8 ± 26.9	0.3888	0.2667
Triglyceride (mg/dL)	147.5 (113.3, 207)	117 (76.3, 152.85)	0.0007	131 (93, 193)	112.5 (89.8, 192.3)	0.1090	0.0383
HDL-C (mg/dL)	50.1 ± 10.6	51.8 ± 13.0	0.4625	52.8 ± 12.5	53.1 ± 11.8	0.9378	0.3083
RLP-C (mg/dL)	5.7 (4.6, 10.9)	3.1 (2.4, 6)	0.0068	6.4 (3.7, 7.5)	4.3 (3.3, 7.5)	0.1224	0.0484
AST (U/L)	20.0 (20.0, 43.5)	29.0 (22.0, 35.5)	0.6009	23.0 (20.0, 39.0)	21.0 (18.0, 34.5)	0.0305	0.5429
ALT (U/L)	36.0 (23.5, 50.0)	25.0 (19.0, 44.0)	0.0291	30.0 (19.0, 46.0)	20.5 (16.0, 42.0)	0.0231	0.2246
GGT (U/L)	46.0 (27.5, 91.5)	30 (18.5, 46.0)	<0.0001	36.0 (23.0, 69.0)	29.0 (19.3, 56.8)	0.0384	0.0020
eGFR (mL/min/1.73m^2^)	80.5 ± 23.3	81.9 ± 20.6	0.5869	75.4 ± 20.9	75.2 ± 19.2	0.8680	0.1466
Uric acid (mg/dL)	6.2 ± 1.7	5.6 ± 1.2	0.0035	5.2 ± 1.4	5.2 ± 1.4	0.7185	0.0081
HMW adiponectin (μg/mL)	1.43 (0.57, 1.90)	1.35 (0.57, 1.96)	0.7143	1.75 (0.76, 2.63)	2.15 (0.76, 3.23)	0.0086	0.0469
Leptin (ng/mL)	19.9 (9.5, 37.4)	17.3 (10.6, 28.1)	0.0062	17.6 (15.6, 32.6)	17.3 (9.9, 30.3)	0.0176	0.2899
sDPP-4/CD26 (ng/mL)	1124±250.4	965.9 ± 21.4	0.0006	1084±381.8	955.3 ± 261	0.0261	0.3758
PAI-1 (ng/mL)	83.7 ± 34.5	70.6 ± 32.7	0.0014	76.0 ± 30.1	57.8 ± 26.1	0.0016	0.6334
PAP (μg/mL)	0.55 (0.50, 0.75)	0.50 (0.40, 0.60)	0.0745	0.60(0.50,0.80)	0.70 (0.50, 0.80)	0.0295	0.0332
CAP (dB/m)	314.9 ± 42.4	325.5 ± 41.9	0.2969	317.6 ± 50.0	299.0 ± 47.5	0.0176	0.0196
LSM (kPa)	4.3 (3.5, 8.8)	4.7 (3.1, 6.1)	0.8991	5.3 (3.5, 8.0)	4.5 (3.6, 6.6)	0.5917	0.5201
FAST score	0.203 (0.089, 0.551)	0.299 (0.133, 0.438)	0.4452	0.195 (0.056,0.521)	0.113 (0.039,0.897)	0.0422	0.0373

Data are mean ± SD or the median and inter-quartile ranges.

BMI, Body Mass Index; VFA, Visceral Adipose Area; SBP, Systolic Blood Pressure; DBP, Diastolic Blood Pressure; FPG, Fasting Plasma Glucose; LDL-C, Low Density Lipoprotein Cholesterol; HDL-C, High Density Lipoprotein Cholesterol; RLP-C, Remnant Like Lipoprotein Cholesterol; AST, Aspartate Aminotransferase; ALT, Alanine Transaminase; GGT, γ-Glutamyltranspeptidase; eGFR, Estimated Glomerular Filtration; HMW, High-Molecular Weight; PAI-1, Plasminogen Activator Inhibitor-1; PAP, Plasmin-α2-Antiplasmin complex; CAP, Controlled Attenuation Parameter; LSM, Liver Stiffness Measurement; FAST, FibroScan-AST.

In the ILI group, body weight and body mass index showed a significant decrease at the end of the treatment period, but these parameters showed no changes in the pemafibrate group (Table 2). The reduction in body weight from baseline to 24 weeks was significantly larger in the ILI group. Serum TG decreased in the pemafibrate group but did not change in the IIL group, and the percentage reduction in TG from baseline to 24 weeks was significantly larger in the pemafibrate group than in the ILI group (Table 2). Serum RLP-C also decreased in the pemafibrate group but did not change in the ILI group, and the percentage reduction in RLP-C from baseline to 24 weeks was significantly larger in the pemafibrate group. Serum sDPP-4/CD26 and leptin were significantly reduced in both groups, and plasma PAI-1 was also reduced in both groups. The VFA showed no significant change in either group (Table 2).

**Fig. 2:**
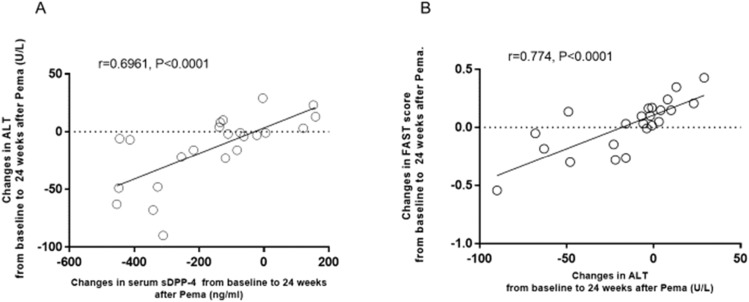
Correlation between changes in ALT and those in serum DPP-4/CD26 (A) or in FAST score (B) after 24 weeks of treatment with pemafibrate (0.2 mg/day) in patients with type 2 diabetes and non-alcoholic fatty liver disease.

In the pemafibrate group, changes in ALT were significantly positively correlated with changes in serum sDPP-4/CD26 (Fig. 2A) and in the FAST score (Fig. 2B). In addition, changes in the FAST score were significantly positively correlated with changes in serum sDPP-4/CD26 in the pemafibrate group (*r* = 0.5169, *p* = 0.0116). In the ILI group, there was no significant correlation between changes in ALT and those in serum sDPP-4/CD26 (*r* = 0.3436, *p* = 0.0857) or FAST score (*r* = 0.3322, *p* = 0.1103).

## Discussion

The present study demonstrated no significant reductions in CAP, LSM, or the FAST score in the pemafibrate group, while CAP was significantly decreased in the ILI group. These results suggested that weight loss through an intensive lifestyle intervention was better than pemafibrate in terms of the main indicators that assess improvement in the pathogenesis of MASLD, since the ILI group, but not the pemafibrate group, improved hepatic steatosis (CAP), FAST score, visceral fat accumulation, and serum HMW adiponectin. Other studies reported that pemafibrate did not reduce either CAP or LSM but did improve the FAST score.^28^^,^^29^ The FAST score is effective for identifying patients at risk of progressive MASH with significant fibrosis.^25^ However, these two studies were retrospective in nature and lacked a control group, so it cannot be concluded that the decrease in the FAST score was specific to pemafibrate and would not occur with other fibrates. Although the authors found no significant reduction in the FAST score with pemafibrate, the present results showed a significant, strong positive correlation between changes in ALT and the FAST score, suggesting that a reduction in liver enzymes by pemafibrate may be associated with an improved FAST score.

The present study confirmed that pemafibrate significantly decreases serum levels of the liver enzymes ALT and GGT. The authors also found that the pemafibrate group and the ILI group observed a comparable and significant reduction in liver enzyme (serum transaminase) levels in people with type 2 diabetes and MASLD. In the ILI group, there was a modest but significant weight loss (∼3 %), while body weight did not change in the pemafibrate group. Although the authors found a modest weight loss in the ILI group, serum transaminase levels and CAP significantly decreased. There is strong evidence that in people with obesity and MASLD, weight loss results in improvement or resolution of MASLD, and that greater weight loss produces a greater response rate.^1^^,^^2^ In a previous study, 50 % of nonobese subjects achieved remission of MASLD with a 3 % to 5 % weight reduction.^30^ In the ILI group, liver enzymes decreased in parallel with body weight and CAP, suggesting that in this group, liver dysfunction may improve by decreasing liver fat (TG) via significant body weight loss. The present findings supported the current recommendation for weight loss using lifestyle modification as the first step in the management of patients with MASLD, including patients with type 2 diabetes. Thus, the present study demonstrated that pemafibrate is as effective as modest weight loss with lifestyle modification for reducing elevated liver enzymes in people with type 2 diabetes and MASLD. Recently, the large-scale Pemafibrate to Reduce Cardiovascular Outcomes by Reducing Triglycerides in Patients with Diabetes (PROMINENT) study also showed that pemafibrate is associated with a lower incidence of MASLD in patients with hypertriglyceridemia.^31^

The mechanisms responsible for the beneficial effects of pemafibrate in MASLD remain to be determined. The authors hypothesized that pemafibrate improves liver dysfunction by decreasing the TG content in the liver via β-oxidation of free fatty acids with activation of PPARα. However, CAP was unchanged after treatment with pemafibrate, and in the pemafibrate group, changes in ALT were not significantly correlated with those in CAP. A previous study also found that liver fat content measured by magnetic resonance imaging-estimated proton density fat fraction did not significantly change from baseline after 72 weeks of treatment with pemafibrate in patients with MASLD.^16^ Similarly, in a study in a NASH mouse model, pemafibrate treatment did not reduce the total liver fat content, but it reduced F4/80-positive macrophage accumulation into the liver, ballooning degeneration of hepatocytes, and improved liver fibrosis, with a reduction in collagen 1α1 mRNA expression in the liver, and also decreased the ALT level and expression of pro-inflammatory genes.^32^ In another mouse model in which MASH was induced by an Amylin liver NASH diet, pemafibrate improved histological findings in the liver, including hepatocyte ballooning and hepatic fibrosis.^33^ These findings suggest that pemafibrate might improve liver fibrosis by alleviating inflammation and/or directly suppressing liver fibrosis. In a clinical study, 1-year pemafibrate treatment improved markers of hepatic function, inflammation, and fibrosis in non-diabetic patients with MASLD.^34^ Improvement of hepatic fibrosis markers was significantly correlated with improvement of hepatic inflammation markers and triglyceride levels.^34^ Another clinical trial showed that pemafibrate treatment did not reduce the liver fat content but did significantly reduce magnetic resonance elastography-based liver stiffness,^29^ suggesting that the reduction in magnetic resonance elastography-based liver stiffness may reflect the amelioration of hepatic fibrosis and lobular inflammation, as evidenced by a reduction in serum liver fibrosis markers. Taken together, these findings indicate that pemafibrate may improve liver function by reducing hepatic inflammation and fibrosis without altering hepatic TG accumulation, although this study showed no significant reduction in LSM.

The present study demonstrated that serum levels of sDPP-4/CD26 significantly decreased after treatment in both the pemafibrate group and the ILI group. Several studies, including ours, reported that weight loss through calorie restriction or the SGLT2 inhibitor dapagliflozin can induce a significant reduction in serum sDPP-4/CD26 in people with type 2 diabetes.^21^^,^^22^ The authors confirmed that weight loss induced a reduction in serum sDPP-4/CD26 levels in the ILI group. The present study also provided the first evidence that pemafibrate decreases the serum level of sDPP-4/CD26 as much as weight loss, together with a significant improvement of liver enzymes (ALT and GGT) in patients with type 2 diabetes and MASLD. In a previous study, the authors found that serum sDPP-4/CD26 correlates positively with the severity of liver fibrosis evaluated by LSM and the FAST score,^35^ suggesting that serum levels of sDPP-4/CD26 may be considered a new biomarker for liver fibrosis and progressive MASH in people with type 2 diabetes. Similar to weight loss, pemafibrate may ameliorate liver dysfunction by reducing serum sDPP-4/CD26 in patients with type 2 diabetes and MASLD. However, this improvement in the pemafibrate group may be independent of reductions in body weight or visceral fat. The authors also found that the change in sDPP-4/CD26 shows a strong positive correlation with the changes in ALT and the FAST score in both the pemafibrate group and the ILI group. Taken together, these results indicate that the main source of sDPP-4/CD26 in these patients may be the liver. In fact, a previous study showed a strong positive correlation between the change in serum sDPP-4/CD26 and changes in ALT or GGT after treatment with the sodium-glucose transport protein 2 inhibitor dapagliflozin.^21^

The present study has some limitations. First, the number of participants was small, so a larger study is needed to confirm these findings, and second, the standard lifestyle recommendations group was not a real control group because it was heterogeneous. The third limitation is a slight reduction in body weight, or BMI, in the ILI group. The fourth limitation is a short study duration to evaluate the effects of pemafibrate on liver steatosis or fibrosis. A further study that includes a liver biopsy is also warranted to understand whether the liver fat content did not change in the pemafibrate group.

In conclusion, mild weight loss through an intensive Lifestyle Intervention (ILI) was better than pemafibrate in terms of the main indicators that assess improvement in the pathogenesis of MASLD, since the ILI group, but not the pemafibrate group, improved hepatic steatosis (CAP), FAST score, visceral fat accumulation, and serum HMW adiponectin. However, pemafibrate 0.2mg/day is as effective as mild weight loss for improvements in liver enzyme and serum sDPP-4/CD26 in people with type 2 diabetes and MASLD. Thus, pemafibrate may be another therapeutic strategy for MASLD in people with type 2 diabetes who undergo lifestyle changes, because its effect is independent of a decrease in body weight. Future larger studies should be needed to confirm the beneficial effects of adding pemafibrate on the resolution of MASLD in people with type 2 diabetes who undergo lifestyle changes.

## Consent to publish declaration

All authors agree to the publication of this study.

## Authors’ contributions

TJ and YA contributed to the study design, data collection, and drafting the manuscript. SS, DT, KS, and KK helped with data collection. TJ, SS, TI, and TT contributed to the discussion and reviewed the manuscript. IU reviewed/edited the manuscript. TJ and YA researched data and wrote and reviewed/edited the manuscript.

## Funding

The present study was not funded.

## Data availability

The datasets generated and/or analyzed during the current study are available from the corresponding author upon reasonable request.

## Declaration of competing interest

The authors declared no potential conflicts of interest with respect to the research, authorship and/or publication of this article.
